# An Intramuscular DNA Vaccine for SARS-CoV-2 Decreases Viral Lung Load but Not Lung Pathology in Syrian Hamsters

**DOI:** 10.3390/microorganisms9051040

**Published:** 2021-05-12

**Authors:** Shanna S. Leventhal, Chad Clancy, Jesse Erasmus, Heinz Feldmann, David W. Hawman

**Affiliations:** 1 Laboratory of Virology, Division of Intramural Research, National Institute of Allergy and Infectious Diseases, National Institutes of Health, Rocky Mountain Laboratories, Hamilton, MT 59840, USA; shanna.leventhal@nih.gov (S.S.L.); chad.clancy@nih.gov (C.C.); feldmannh@niaid.nih.gov (H.F.); 2 Rocky Mountain Veterinary Branch, Division of Intramural Research, National Institute of Allergy and Infectious Diseases, National Institutes of Health, Rocky Mountain Laboratories, Hamilton, MT 59840, USA; 3 Department of Microbiology, University of Washington School of Medicine, Seattle, WA 98195, USA; jerasmus@uw.edu; 4 HDT Bio, Seattle, WA 98102, USA

**Keywords:** SARS-CoV-2, syrian hamster model, DNA Vaccine

## Abstract

The 2019 novel coronavirus, SARS-CoV-2, first reported in December 2019, has infected over 102 million people around the world as of February 2021 and thus calls for rapid development of safe and effective interventions, namely vaccines. In our study, we evaluated a DNA vaccine against SARS-CoV-2 in the Syrian hamster model. Hamsters were vaccinated with a DNA-plasmid encoding the SARS-CoV-2 full length spike open reading frame (ORF) to induce host cells to produce spike protein and protective immune responses before exposure to infectious virus. We tested this vaccine candidate by both intranasal (IN) and intramuscular (IM) routes of administration and complexing with and without an in vivo delivery reagent. Hamsters receiving prime-boost-boost IM-only vaccinations recovered body weight quicker, had decreased lung viral loads, and increased SARS-CoV-2-specific antibody titers compared to control vaccinated animals but, surprisingly, lung pathology was as severe as sham vaccinated controls. The IM/IN combination group showed no efficacy in reducing lung virus titers or pathology. With increasing public health need for rapid and effective interventions, our data demonstrate that in some vaccine contexts, significant antibody responses and decreased viral loads may not be sufficient to prevent lung pathology.

## 1. Introduction

Severe acute respiratory syndrome coronavirus 2 (SARS-CoV-2), first reported in December 2019, emerged as a highly transmissible and rapidly spreading disease. It was defined as a pandemic by the World Health Organization (WHO) within 4 months of its appearance [1] and has infected over 102 million people around the world as of February 2021 [2]. The disease it causes, coronavirus disease 2019 (COVID-19), in general can be characterized by symptoms of pneumonia including fever, cough, and fatigue but also may induce cytokine storm syndrome which causes severe respiratory failure or distress and this is considered the main cause of death in patients with COVID-19 [1]. Like SARS-CoV-1, SARS-CoV-2 vaccine approaches have focused on utilizing the viral spike protein to elicit protective immune responses [1,3]. The spike protein covers the surface of the virus and binds to human host cell receptor angiotensin-converting enzyme 2 (ACE-2) to mediate viral cell entry [4], making it key to infection and thus a good target for neutralizing antibodies and protective immunity [5]. Several vaccines are already being developed, entering clinical trials, or being released to the public on emergency use authorization, including various subunit and nucleic acid vaccines that make use of the spike protein itself or the spike gene sequence [3,6,7]. Out of these, only one DNA vaccine, INO-4800 by Inovio/ Beijing Advaccine Biotechnology Company [8], has reached clinical trials. Although there are no DNA vaccines currently licensed for use in humans, hundreds of preclinical and clinical trials have shown promising immunogenicity and reliable safety data, especially against viral infections and even cancer [9]. DNA vaccines are less expensive, easy to develop, focus immune responses on an antigen of interest, and have increased stability for storage and shipping [10], all of which can be beneficial characteristics for widespread production. Here we report a DNA vaccine strategy evaluated in two study arms, prime-boost and prime-boost-boost, where we compared immune response and protective efficacy of intramuscular (IM), intranasal (IN), or combination IM/IN vaccinations with or without an in vivo delivery reagent in a Syrian hamster SARS-CoV-2 disease model. The in vivo-jetPEI delivery transfection reagent used here is a ~22 kDa polyethyleneimine (PEI)-based cationic nanoparticle reagent that has been shown to enhance delivery of nucleic acids including DNA [11,12]. Currently, in vivo-jetPEI is an attractive candidate for SARS-CoV-2 intervention and is used here due to its increased transfection efficiency in multiple tissues, decreased toxicity, and availability for animal studies. Further, PEI-conjugated DNA has been previously evaluated as a vaccine candidate for SARS-CoV-1 [13] and PEI-mediated delivery of DNA to lung tissue can result in efficient antigen expression [14].

The Syrian hamster model is highly susceptible to SARS-CoV-2, with an ID50 of 5 TCID_50_ [15]. The hamster ACE-2 receptor consistently binds to the SARS-CoV-2 spike receptor binding domain (RBD) at levels higher than human ACE-2 [15]. Previous characterizations of this model showed that infected hamsters present with clinical disease around 3 days post IN infection and develop moderate to severe broncho-interstitial pneumonia and prolonged viral shedding out to at least 10 days post infection in the upper respiratory tract [15]. While the lungs are the major site of viral replication, viral gRNA was detected in multiple tissues [15]. Overall, infected hamsters display a transient disease characterized by high viral loads in the lung, viral shedding, and moderate to severe lung pathology [15], giving clear clinical, pathologic, and virologic criteria on which to evaluate vaccine efficacy. While DNA vaccines are more commonly delivered via the IM route [9], we evaluated IN and IM/IN combinations to explore the potential of inducing mucosal immunity and thus preventing SARS-CoV-2 primary infection or transmission through the proposed natural route of infection–nasal inhalation [1].

## 2. Materials and Methods

### 2.1. Biosafety and Ethics Statement

All work with SARS-CoV-2 was done following guidelines put forth by the Institutional Biosafety Committee (IBC) in biocontainment level 3 at the Rocky Mountain Laboratories, NIAID, NIH, Hamilton, MT. Sample removal from biocontainment followed approved and published protocols [16]. All animal work was approved by the Institutional Animal Care and Use Committee (Protocol #2020-51, approved 6 February 2021) on site and performed in accordance with the recommendations described in the Guide for the Care and Use of Laboratory Animals of the National Institutes of Health, the Office of Animal Welfare, the United States Department of Agriculture in an association for Assessment and Accreditation of Laboratory Animal Care-Accredited Facility. Animals were group housed in HEPA-filter cage systems enriched with nesting material. Commercial food and water were available ad libitum.

### 2.2. SARS-CoV-2 Viral Stock

SARS-CoV-2 strain nCoV-WA1-2020 (MN985325.1) was provided by the CDC, Atlanta, USA and propagated at RML in Vero E6 cells in DMEM supplemented with 2% fetal bovine serum, 1 mM L-glutamine, 50 µg/mL penicillin, and 50 µg/mL streptomycin. Vero E6 cells were maintained in DMEM supplemented with 10% fetal bovine serum, 1 mM L-glutamine, 50 U/mL penicillin, and 50 µg/mL streptomycin. The virus stock was free of contaminants and was confirmed to have identical sequence to original strain published in Genbank.

### 2.3. Generation of Vaccine Plasmid

**Figure A1:**
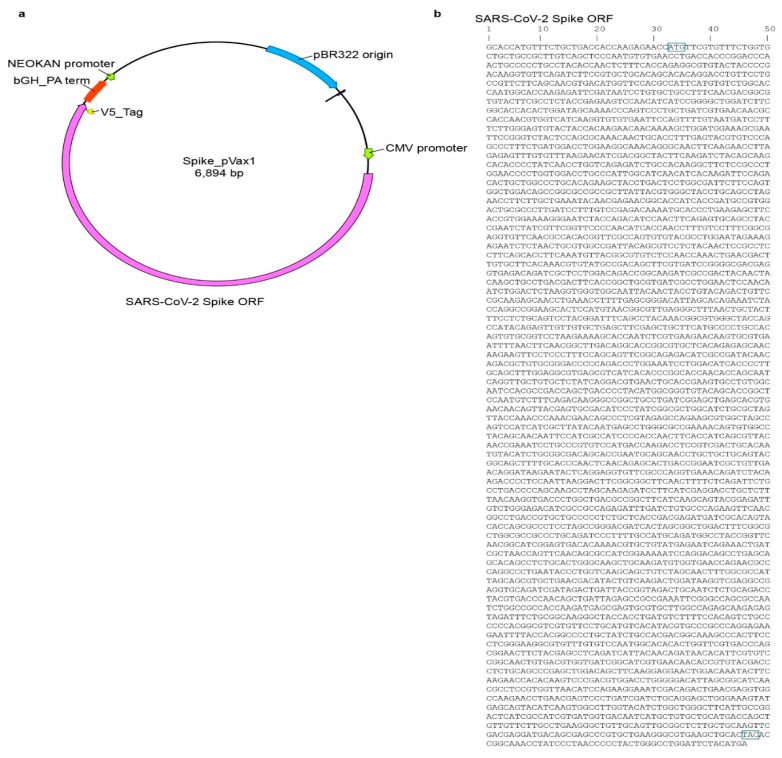
Sp_pVax1 Plasmid Map and Codon-Optimized SARS-CoV-2 Spike Sequence. (**a**) Spike_pVax1 plasmid map showing SARS-CoV-2 spike ORF with V5 epitope tag inserted into the pVax1 vector backbone and (**b**) codon-optimized spike sequence. The spike open reading frame start codon (ATG) and final codon (TAC) are indicated in blue boxes, the V5 epitope tag sequence immediately follows that of the spike and ends in final stop codon.

Sp_pVax1 plasmid was constructed using a codon-optimized SARS-CoV-2 full length spike based on SARS-CoV-2 isolate Wuhan-Hu-1 (Genbank MN908947.3) with a 3′ V5 epitope tag added at the nucleotide level between the 3′ end of the spike sequence and final stop codon. The final sequence was commercially synthesized (Genscript, Piscataway, NJ, USA) and inserted into the pVax1 vector (ThermoFisher, Waltham, MA, USA) via standard in vitro techniques. The plasmid map as well as the spike sequence are provided in Appendix A Figure A1. For animal studies, plasmid was grown up in DH5a competent cells (ThermoFisher, Waltham, MA, USA) and purified using Qiagen EndoFree Plasmid Mega Kit. Final plasmid stock was verified via sequencing by the Rocky Mountain Laboratory Genomics Core.

### 2.4. Syrian Hamsters

Syrian hamsters (*Mesocricetus auratus*) of >5 weeks of age, male and female, were purchased commercially (Charles River, Wilmington, MA, USA) and housed with food and water ad libitum. For all procedures and sample collection, hamsters were anesthetized by inhalation of vaporized isoflurane.

### 2.5. Immunofluorescence Assay (IFA) and Western Blot

BHK cells (ATCC, Manassas, VA, USA) were plated in a 12-well at 100,000 cells/mL for ~80–90% confluency at time of transfection. Sp_pVax1 and empty pVax1 plasmids were transfected into separate wells using Mirusbio TransIT-LT1 Transfection Reagent and recommended protocol. After 24 h, for IFA, cells were washed, permeabilized, and incubated with mouse anti-V5 epitope antibodies (ThermoFisher) at 1:500 for 1 h, washed, and incubated with secondary Alexa Fluor 488-conjugated anti-mouse antibody (ThermoFisher) at 1:2000 for 1 h before final wash and imaging under Biorad ZOE fluorescent cell imager. For western blot, cell lysate was collected using Thermofisher RIPA Lysis and Extraction Buffer and Roche c0mplete protease-inhibitor tablets, and recommended protocols. 15 μL sample was mixed with 15 μL prepared Laemlli buffer (10% SDS, 0.1 M DTT), heated for 10 min at 99 °C, ice 3 min, spin 17,000× *g* 2 min, loaded onto 12-well Biorad Criterion TGX precast Gels 10% and run at 150 V for 1 h using Biorad PowerPac HC alongside BioRad Precision Plus Protein Standards Dual Color Ladder. Proteins were transferred to a nitrocellulose membrane using Biorad Trans-Blot Turbo Transfer Pack (midi) and Trans-Blot Turbo Transfer System for 30 min followed by incubation in PBS-1% Tween 5% milk block (blocking buffer) overnight. Next day, the blot was incubated in Invitrogen primary antibody mouse anti-V5 at 1:5000 diluted in blocking buffer for 1 h followed by wash and incubation with secondary antibody anti-mouse HRP (Jackson Immunoresearch) 1:10,000 diluted in blocking buffer for 1 h. Blot was then washed and imaged using supersignal west pico PLUS chemiluminescent substrate (Fisher Scientific, Waltham, MA, USA) and Proteinsimple FluorChem E Imager.

### 2.6. Preparation of Vaccine

Saline vaccinations were prepared by diluting 100 µg of plasmid DNA in sterile DPBS solution. Complexed vaccinations were prepared by complexing 25 µg of DNA to in vivo-jetPEI (Polyplus Transfection, New York, NY, USA) following recommended protocol, scaled up to make a stock for all complexed vaccinations. Briefly, DNA plasmid and in vivo-jetPEI were diluted in provided 10% glucose solution before mixing and incubation with reagent.

### 2.7. RNA Extraction and Sub-Genomic E (SgE) Quantitative Reverse-Transcription Polymerase Chain Reaction (qRT-PCR)

RNA was extracted from oral and rectal swabs using the Qiagen RNA-mini isolation kit and provided protocol while RNA from organ tissue was isolated via Qiagen RNeasy mini isolation kit and provided protocol. Viral RNA was quantified via qRT-PCR using Qiagen Quantifast one-step qRT-PCR master mix, primers corresponding to SgE SARS-CoV-2 RNA segment (IDT) [17], and run on a Quantstudio 5 RT-PCR system (ThermoFisher). Cycling conditions were as follows: initial hold of 50 °C for 10 min, initial denaturation of 95 °C for 5 min, and 40 cycles of 95 °C for 15 s followed by 60 °C 30 s. SARS-CoV-2 RNA standards with known copy number were prepared in house, diluted, and run alongside samples for quantification.

### 2.8. Enzyme-Linked Immunosorbent Assay (ELISA)

An in-house ELISA was used for evaluation of anti-RBD antibodies in vaccinated hamster’s sera. 96-well flat bottom Immuno Plate MaxiSorp (ThermoFisher) were incubated overnight with SARS-CoV-2 RBD antigen (Genscript) diluted in DPBS at a concentration of 50 ng protein/well. Next day plates were blocked with 1×PBS–0.05% Tween–5% milk (blocking buffer) and incubated with hamster sera serially diluted 1:4 starting at a 1:100 dilution in blocking buffer for 1 h. Plate was washed and blocked with secondary Affinity Purified Antibody Peroxidase Labeled Goat anti-Hamster IgG(H + L) (KPL) for another hour. Afterwards, plate was washed and incubated with SeraCare KPL ABTS Peroxidase Substrate System (2-Component) for 15 min before 5% SDS in water was applied. 405 nm absorbance was measured by BioTek Imager.

### 2.9. TCID_50_ Assay

Lung samples were weighed and then homogenized in 1 mL DMEM supplemented with 2% fetal bovine serum, 1 mM L-glutamine, 50 U/mL penicillin, and 50 µg/mL streptomycin with sterile bead. Sample was diluted down a 10-fold gradient and 100 uL of each dilution was transferred to Vero UNC cells. Vero UNC were plated day prior at 10,000 cells/well in a 96-well plate for ~80–90% confluency at time of lung homogenate supernatant transfer. Vero UNC were then incubated with homogenate samples for 6 days before CPE was read. Titers normalized to mg of tissue. TCID_50_ was calculated using the Reed and Muench method.

### 2.10. Neutralization Assay

Sera samples were inactivated at 56 °C for 30 min and serially diluted 1:2 starting at a 1:10 dilution in infection media (DMEM supplemented with 2% fetal bovine serum, 1 mM L-glutamine, 50 U/mL penicillin, and 50 µg/mL streptomycin). SARS-CoV-2 stock identical to that used for infection was diluted to contain 120 TCID_50_ and was added 1:1 to each dilution well. The virus-sera mixture was incubated at 37 °C for 1 h and then added to Vero UNC cells plated day prior in a 96-well flat bottom plate at 10,000 cells/well for ~80–90% confluency at time of initiating neutralization assay. Cells were incubated at 37 °C and checked for serum toxicity after 24 h and CPE after 6 days. The viral neutralization titer is reported as the inverse of the last dilution of antibody where CPE is not observed.

### 2.11. Histology

Tissues were fixed in 10% neutral buffered formalin with two changes, for a minimum of 7 days before processing. Tissues were placed in cassettes and processed with a Sakura VIP-6 Tissue Tek, on a 12-h automated schedule, using a graded series of ethanol, xylene, and ParaPlast Extra. Embedded tissues are sectioned at 5 µm and dried overnight at 42 degrees C prior to staining. Specific anti-CoV immunoreactivity was detected using Sino Biological Inc, Beijing, China. SARS-CoV/SARS-CoV-2 nucleocapsid antibody (Sino Biological cat#40143-MM05) at a 1:1000 dilution. The secondary antibody was the Vector Laboratories ImPress VR anti-mouse IgG polymer (cat# MP-7422). The tissues were then processed for immunohistochemistry using the Discovery Ultra automated stainer (Ventana Medical Systems) with a ChromoMap DAB kit (Roche Tissue Diagnostics cat#760–159).

### 2.12. Statistical Analyses

2-way ANOVA and Kruskal-Wallis statistical tests were done using GraphPad Prism 8 software. Experiments were performed once with *n* = 6 per group for prime-boost and *n* = 12 for prime-boost-boost in spike vaccinated groups. For control groups where hamsters received sham vaccination, *n* = 12 for prime-boost and *n*= 6 for prime-boost-boost.

## 3. Results

### 3.1. SARS-CoV-2 Spike Protein Is Expressed from the pVax1 DNA Vector Backbone

**Figure 1:**
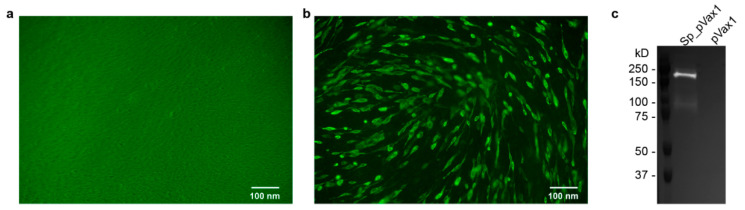
Sp_pVax1 and pVax1 Plasmid Expression. Immunofluorescence assay (IFA) on BHK-21 cells transfected with (**a**) pVax1 or (**b**) Sp_pVax1. Cells were permeabilized and probed with primary anti-V5 antibody and secondary fluorescent antibody at 24 h post transfection to visualize spike protein-positive cells. BHK cell lysate was collected at 24 h post transfection and evaluated via (**c**) western blot to detect appropriately sized spike protein at 180–200 kDa (upper bright band) and cleavage product (lower light band).

To effectively deliver spike DNA through vaccination, we utilized a plasmid platform consisting of a codon optimized, full length SARS-CoV-2 spike open reading frame (ORF) inserted into the pVax1 vector backbone (Figure A1a,b). In addition, a V5 epitope tag sequence (GKPIPNPLLGLDST) was added to the 3′ end of the spike protein sequence prior to the stop codon (Appendix A Figure A1a,b) to facilitate confirmation of spike protein production in vitro. To confirm expression, the plasmid (Sp_pVax1) was transfected into BHK-21 cells. These cells were analyzed via immunofluorescence assay (IFA) and western blot using an anti-V5 antibody. Compared to cells transfected with empty pVax1 plasmid, which showed no expression, both tests showed efficient spike production. Staining of Sp_pVax1 transfected cells with anti-V5 antibody showed increased levels of fluorescence compared to empty pVax1 transfected cells indicating translation of the spike protein (Figure 1a,b). By western blot of cell lysates, cells transfected with Sp_pVax1 exhibited a band between 150 kD and 250 kD (Figure 1c), consistent with full length in vitro production of the spike protein which has a predicted size of 180–200 kDa [5].

### 3.2. Prime-Boost Does Not Affect COVID-19 Disease Characteristics but Shows a Trending Improvement Compared to Controls for All Groups

**Figure 2:**
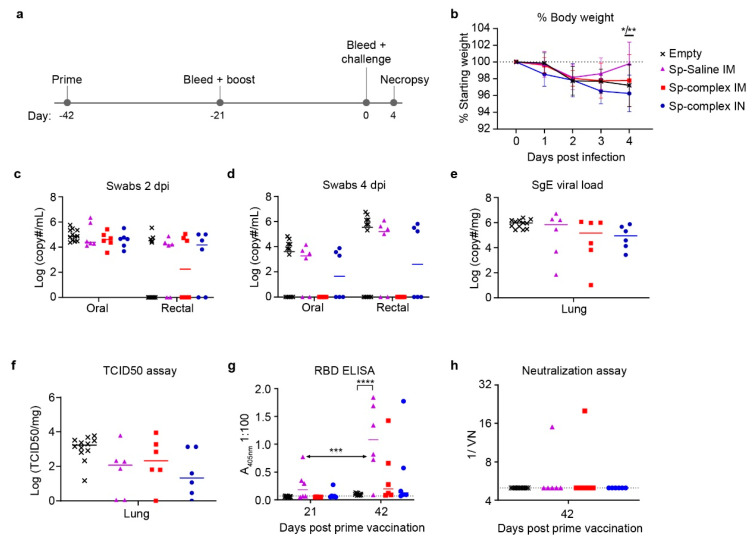
Prime-Boost Hamster Body Weight, Viral Loads, and Antibody Characterization. Prime-boost hamsters were divided into three spike-vaccinated groups receiving Sp_pVax1 IM vaccination of 100 µg DNA diluted in saline (Sp-Saline IM), IM vaccination of 50 µg Sp_pVax1 DNA complexed to in vivo delivery reagent (Sp-complex IM), and IN vaccination of 50 µg Sp_pVax1 DNA complexed to in vivo delivery reagent (Sp-complex IN). *n* = 12 for empty pVax1 group and *n* = 6 for Sp-vaccinated groups. (**a**) 3-weeks post prime, hamsters received boost and 3-weeks later were challenged IN with 1000 TCID_50_ of SARS-CoV-2. (**b**) Animals were weighed daily to monitor weight loss, detailed weight loss for individual animals can be found in Appendix A Figure A2. (**c**,**d**) Viral shedding was measured in nasal and rectal swabs at (**c**) day 2 and (**d**) 4 PI. Viral loads in the lungs at day 4 PI were measured by (**e**) qRT-PCR and (**f**) TCID_50_. Antibody titers to the SARS-CoV-2 spike RBD were evaluated by (**g**) ELISA and (**h**) serum neutralizing capacity against infectious SARS-CoV-2 measured. Statistical analyses were done using Kruskal Wallis and 2-way ANOVA. * *p* < 0.05, ** *p* < 0.01, *** *p* < 0.001, **** *p* < 0.0001.

After confirming in vitro expression of the SARS-CoV-2 spike we next evaluated the vaccine in protection against virus challenge in the Syrian hamster disease model [15] following a prime-boost regimen. Hamsters were divided into three spike-vaccinated groups and one non-spike vaccinated control group (Empty). Hamsters received either Sp_pVax1 via IM injections with 100 µg of DNA diluted in saline (Sp-Saline IM), IM with 50 µg of DNA complexed to in vivo delivery reagent (Sp-complex IM), or IN with 50 µg of DNA complexed to in vivo delivery reagent (Sp-complex IN). Lower quantities of DNA were delivered with the in vivo reagent compared to the Sp-saline IM group due to maximum injection volumes and animal welfare considerations. Control hamsters received any one of the three spike-vaccination formulations with empty pVax1 and were pooled for analyses. An identical boost vaccination was given 3 weeks post-prime, and hamsters were challenged IN three weeks post-boost with 1000 TCID_50_ of SARS-CoV-2 (ID_50_ = 5 TCID_50_ [15]) (Figure 2a). As the SARS-CoV-2 hamster model is not lethal [15], we performed a timed necropsy on 4 days post infection (dpi).

In a comparison of weight loss over the course of infection, all groups of hamsters initially lost weight and only the Sp-Saline IM group of hamsters began to recover on day 3 with slight but significant increase in weight compared to both controls and the IN group by 4 dpi (Figure 2b). However, in all groups weight loss was mild (<5%). Oral swabs at 2 dpi showed similar viral loads for the vaccinated groups compared to controls (Figure 2c). Viral loads in rectal swabs at 2 dpi and both oral and rectal swabs at 4 dpi, for most hamsters were below the limit of detection (LOD) of our sub-genomic (SgE) PCR (Figure 2c,d). We next evaluated lung viral loads, both by SgE qRT-PCR and infectious virus TCID_50_ assay. No vaccine group showed significant decrease in viral loads, with only a trend towards lower RNA loads and infectious virus in Sp-vaccinated animals compared to controls (Figure 2e,f), suggesting this vaccination regimen did not significantly impact viral replication within the lungs.

Lastly, we evaluated the humoral response to vaccination by SARS-CoV-2 RBD ELISA three-weeks after prime-vaccination (day 21) and three-weeks after boosting (day 42) prior to challenge. Surprisingly, compared to control vaccinated animals neither the Sp-complex IM nor Sp-complex IN groups had significantly increased anti-RBD titers at D42 (Figure 2g). Only the saline IM group had statistically increased anti-RBD titers compared to control vaccinated animals (Figure 2g). However, across all groups, anti-RBD ELISA titers were low and non-neutralizing (Figure 2h), consistent with our observed lack of protection against virus challenge in spike-vaccinated groups. These data suggest a prime-boost vaccination scheme was insufficient to elicit significant antibody responses to SARS-CoV-2. Given the low immunogenicity of the prime-boost regimen we hypothesized that additional boosting may be necessary to achieve significant protection against SARS-CoV-2 challenge.

### 3.3. Prime-Boost-Boost Vaccination Confers Significantly Decreased Viral Loads against SARS-CoV-2 Challenge

**Figure 3:**
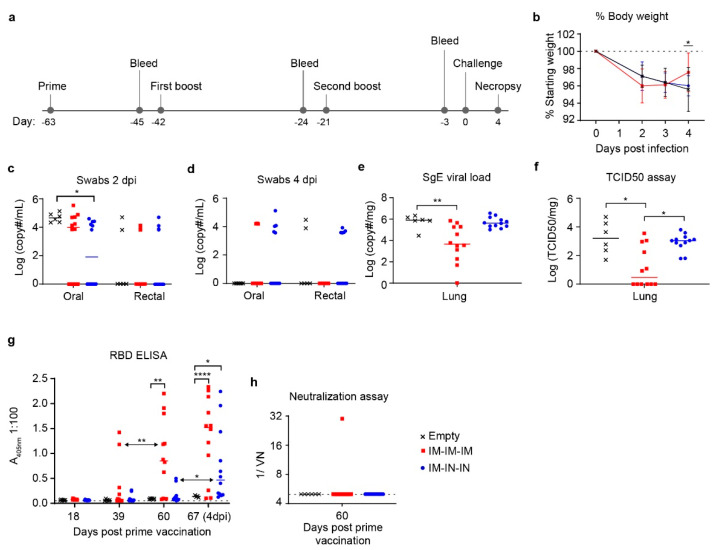
Prime-Boost-Boost Hamster Body Weight, Viral Loads, and Antibody Characterization. Prime-Boost-Boost hamsters were divided into two spike-vaccinated groups receiving IM-IM-IM or IM-IN-IN vaccinations of 50 µg Sp_pVax1 DNA plasmid complexed to in vivo delivery reagent. *n* = 12 per group (**a**) 3-weeks post prime, hamsters received boost, with second boost 3 weeks post first boost, and 3-weeks later were challenged IN with 1000 TCID_50_ of SARS-CoV-2 for four days. Vaccine efficacy was evaluated via comparison of spike-vaccinated and control (**b**) weight over course of infection, detailed weight loss for individual animals can be found in Appendix A Figure A3, (**c**) oral/rectal swabs at 2 dpi, (**d**) oral/rectal swabs at 4 dpi, (**e**) lung viral loads by SgE PCR at 4 dpi, (**f**) lung infectious virus TCID_50_ at 4 dpi, (**g**) RBD antibody titers by ELISA at D18 (three weeks post prime), D39 (three weeks post first boost), D60 (three weeks post second boost), and D67 (final day of infection), and (**h**) neutralization assay of sera from D60. Statistical analyses were done using Kruskal Wallis and 2-way ANOVA where * *p* < 0.05, ** *p* < 0.01, **** *p* < 0.0001.

Due to the low immunogenicity of our prime-boost regimen, we next evaluated whether an additional boost would improve immune responses to the vaccine. Prime-boost-boost hamsters were divided into two spike-vaccinated groups and one control group. We elected to perform a prime-boost-boost vaccine regimen solely with DNA complexed to the in vivo delivery reagent as the complexed vaccine showed the greatest trend towards reduced viral titers in the swabs and lung tissue (Figure 2c–f). Spike-vaccinated hamsters received 50 µg of Sp_pVax1 complexed to in vivo delivery reagent in either an IM-prime IM-IM-boosts (IM-only) or an IM-prime IN-IN-boosts (IM/IN combination) schedule with each vaccination separated by 3 weeks (Figure 3a). We evaluated both IM-only and combined IM/IN vaccinations as we hypothesized that IM-priming followed by IN-boosting would elicit greater protective immunity at the site of SARS-CoV-2 exposure (nasal mucosa) and replication (lungs). Control group hamsters (Empty) received either of the two vaccination schedules with 50 µg empty pVax1 complexed to in vivo delivery reagent and were pooled for analyses. Hamsters were challenged three weeks post second boost with 1000 TCID_50_ SARS-CoV-2 and necropsied on 4 dpi as before (Figure 3a). Similar to the prime-boost hamsters, all groups initially lost weight after infection however, by 4 dpi, the IM-only group had slightly but significantly increased weight compared to controls (Figure 3b). Again, weight loss in any group was minimal. At 2 dpi, oral shedding of virus was slightly but significantly reduced in the IM/IN combination group compared to controls (Figure 3c). However, by 4 dpi, the swabs no longer showed significant differences between vaccination routes as most hamster’s viral loads dropped below the SgE PCR limit of detection (Figure 3d). In respect to lung viral loads at 4 dpi, hamsters receiving IM-only vaccination had significantly reduced viral loads by both SgE qRT-PCR (~2 log decrease, *p* = 0.0061) and infectious virus TCID_50_ (~3 log decrease, *p* = 0.0326) compared to the control group (Figure 3e,f). Notably, half of the hamsters in the IM-only group had no detectable infectious virus whereas all hamsters in the control and IM/IN group had detectable infectious virus (Figure 3f).

We next evaluated antibody responses by RBD ELISA. Sera for all groups were analyzed at 18-days after each vaccination (D18, D39, D60) and at D67, four days after infection. By day 60 in the IM-only group, most hamsters had detectable anti-RBD antibody and as a group this increase was statistically significant compared to day 60 controls (Figure 3g). Furthermore, antibody titers in the IM-only group significantly increased from days 39 to 60 demonstrating that the second boost significantly increased anti-RBD titers (Figure 3g). Although the IM/IN combination group did not have significant anti-RBD serum titers prior to infection, after SARS-CoV-2 challenge this group demonstrated a significant anamnestic response to the infection (Figure 3g). Unexpectedly, although our ELISA results demonstrated a significant response against the SARS-CoV-2 RBD, the primary target of neutralizing antibodies, after prime-boost-boost in the IM-only group, serum at this timepoint was non-neutralizing (Figure 3h). These results indicate that although the IM-only vaccination elicited significant RBD-specific antibodies, these antibodies were non neutralizing.

### 3.4. Lung Pathology in Vaccinated Hamsters

Given the significantly reduced viral loads observed in the IM-only prime-boost-boost group, hamster lung tissues were evaluated for histopathology and viral antigen by immunohistochemistry. Unexpectedly, with the exception of a single hamster in the IM-only spike-vaccinated group, classic lesions of SARS-CoV-2 infection in the Syrian hamster model were observed in all evaluated animals (Table 1).

**Figure 4:**
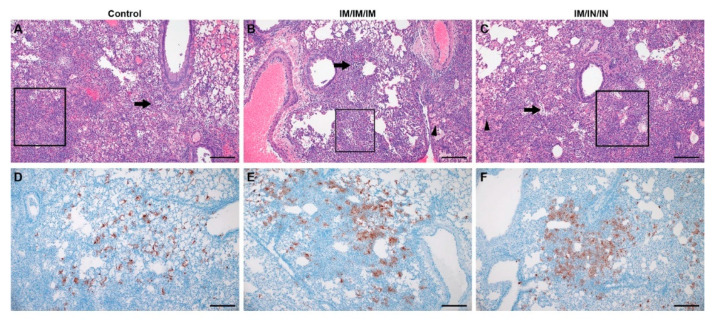
Histopathology and Immunohistochemistry of Control and Vaccinated Prime-Boost-Boost Hamster Lungs. Histopathology of control and vaccinated hamsters (**A**–**C**) are similar and characterized foci of moderate to severe interstitial pneumonia and disruption of loss of pulmonary architecture (boxes), regions of alveolar cellular exudate (arrows) and occasional foci of pulmonary edema (arrowhead; (**B**,**C**)) are indicated. Immunohistochemistry of control and vaccinated hamsters is similar (**D**–**F**) with abundant SARS-CoV-2 immunoreactivity observed in type I and II pneumocytes and macrophages. Representative images for each group are shown. (Bar = 200 μm).

A pronounced bronchiolitis with single cell necrosis and infiltration of leukocytes, primarily neutrophils and macrophages, was noted in all spike-vaccinated groups. However, bronchiolitis tended to be less pronounced in the IM-only spike-vaccinated group with ~50% of evaluated animals having moderate bronchiolitis and 25% having only a mild bronchiolitis (Table 1). Interstitial pneumonia was noted in all spike-vaccinated and control groups but was most severe in the control and IM/IN combination groups. Severe interstitial pneumonia characterized by complete loss of pulmonary architecture, influx of numerous degenerate and non-degenerate neutrophils, presence of abundant pulmonary edema and/or fibrin and pronounced vasculitis was evident in all groups (Figure 4A–C). There was a mild increase in pathology severity in the IM/IN combination group relative to the control hamsters. Although this change may suggest a trend towards an immune enhanced disease state, outside of the mild increase in pathology severity score, the histopathologic changes were identical between control, IM-only and IM/IN combination vaccinated groups. Importantly, there was no evidence of eosinophilic infiltrates, increased mucous production, or smooth muscle hyperplasia that would indicate a hypersensitivity response due to SARS-CoV-2 infection post vaccination. Immunohistochemistry to detect viral antigen showed similar immunoreactivity to viral antigen in pulmonary tissue across groups (Figure 4D–F). Histopathological findings are summarized in Table 1. Cumulatively, our histology and immunohistochemistry findings demonstrate that although IM-only vaccination resulted in significantly reduced viral loads within lung tissue, it did not protect against infection-mediated pathology.

## 4. Discussion

With the ongoing SARS-CoV-2 pandemic and continued rise in COVID-19 case numbers, multiple vaccines are under evaluation for prevention against disease. Among the potential vaccine platforms, DNA candidates have several advantages as they are less expensive, easily developed, and stable for shipping and storage across the globe, all of which are appealing characteristics for dissemination of interventions during a pandemic [9]. The pVax1 backbone has been used in multiple published vaccine investigations and was designed specifically for DNA vaccine development [18]. The plasmid itself is only 3.0 kb with limited eukaryotic DNA sequences to minimize any possibility of chromosomal integration [18]. Further, pVax1 features a kanamycin resistance gene for selection to avoid allergic responses associated with ampicillin as well as a human CMV promoter and BGH polyadenylation signal for efficient DNA expression and mRNA transcription termination, respectively [18]. Recently, the vector has been used in platforms showing enhanced protection from Japanese Encephalitis Virus, influenza H9N2, Dengue virus, Crimean-Congo Hemorrhagic Fever Virus, and anti-tumor applications, [19,20,21,22,23]. These DNA vaccines were able to elicit high titers of protective antibody [19,20,21,22] suggesting this approach may also be effective for COVID-19. Importantly, none of these studies reported negative side effects associated with use of the pVax1 vector [19,20,21,22]. As a major concern of vaccine production is safety, the use of pVax1 and its positive history in vaccine investigation was an important aspect of our plasmid design.

Since DNA vaccines often require boosting [9], we investigated prime-boost and prime-boost-boost schedules in tandem in the Syrian hamster challenge model. This challenge model has been well characterized by our group and recapitulates several aspects of SARS-CoV-2 clinical disease including high viral replication in lung tissue, viral shedding in oral and rectal swabs and moderate to severe pathology in the lung [15]. Challenge in our study was done IN to mimic natural infection [1]. In a study of SARS-CoV-2 hospitalized patients, viral load and shedding were shown to be accurate indicators of increasing COVID-19 disease progression and severity [24,25]. Furthermore, reduced viral shedding in vaccinated groups is desirable to interrupt viral spread to naïve individuals. For all study arms, we surveyed oral/rectal swabs at 2 dpi and 4 dpi to evaluate vaccine-mediated effects on viral shedding. With IN (prime-boost) and IM/IN (prime-boost-boost) vaccinations, we hoped to achieve reduced viral replication in the oral/nasal passage by applying the vaccine to this site and inducing localized mucosal immunity.

The prime-boost regimen evaluated an IM vaccine with DNA diluted in saline compared to both an IM and an IN vaccination with DNA complexed to the PEI-based reagent in vivo-jetPEI. PEI is a cationic polymer that has been investigated for enhancing delivery of nucleic acid vaccines, and IN-delivered PEI-complexed DNA was found to elicit humoral and cellular immunity to SARS-CoV-1 in mice [13]. Other PEI interventions have evaluated higher molecular weight complexes and although these have improved transfection efficiencies, complexes are rapidly removed by macrophages, causing acute toxicity by accumulation of nanoparticles in organs such as the liver and lungs where these immune cells are common [11]. Further studies showed that addition of polyethylene glycol (PEG) to these complexes resulted in limited uptake of nanoparticles by immune cells and thus reduced toxicity in healthy tissues. However, transfection efficiency then only increased in primary tumor cells and actually decreased in tissues such as the lung [11]. Nanomedicine is an actively improving area of research and these methods have been used for other infectious disease treatments with minimal collateral damage to uninfected cells but have yet to be fully developed for application against SARS-CoV-2 [26]. In our study, while neither the saline or PEI-complexed DNA vaccinated animals significantly decreased viral shedding or viral loads, there was a trending decrease in all three vaccination groups compared to controls. Consistent with the similar viral loads across control and spike vaccinated groups, our ELISA and neutralization assay data suggested that our prime-boost vaccine regimen was poorly immunogenic, even when the vaccine was complexed to PEI.

We therefore evaluated whether an additional boost would improve viral control. The prime-boost-boost schedule included both IM and IN vaccinations to compare vaccination routes and again attempt to induce localized immunity in respiratory tissue. Indeed, the IM/IN combination group had a slightly, but significantly, lower average viral load in the oral swabs at 2 dpi compared to controls suggesting this vaccine regimen reduced early viral shedding. However, it did not significantly reduce viral loads within the lungs nor protect against SARS-CoV-2 induced pathology suggesting it did not confer protection against lower respiratory infection with SARS-CoV-2. As lower respiratory disease is associated with severe cases of COVID-19 [27], it is likely critical for potential vaccines to reduce viral loads within the lung tissue to protect against severe disease.

In contrast, although the prime-boost-boost IM-only group did not have significantly reduced viral shedding in oral/rectal swabs, this vaccine regimen did significantly lower viral loads in the lungs by both SgE qRT-PCR and infectious virus titration. Notably, half of the hamsters in this group had no infectious virus in the lungs. Unexpectedly, despite reduced lung viral titers in the IM-only group, histological examination of the lungs showed no improvement in lung pathology. Our data suggest that decreasing viral replication within the lung tissue may not strictly correlate with reduced lung pathology. We also observed slightly exacerbated pathology in the lungs of the hamsters receiving the IM/IN vaccinations relative to the IM-only vaccination or control vaccinated hamsters, indicating that IN vaccination does not confer protection against COVID-19 disease. Similar to our data, non-human primates vaccinated with a vesicular stomatitis virus expressing the SARS-CoV-2 spike were protected following IM but not IN vaccination [28]. Interestingly, these IN vaccinated animals showed limited immune response to the vaccine and exhibited exacerbated COVID-19 pneumonia with evidence of immunopathology [28]. Similar evidence of immunopathology has not been reported in human trials and our data is not supportive of an immune-enhanced disease state. Furthermore, IN delivered vaccines can induce protective immunity to SARS-CoV-1 and SARS-CoV-2 in mice and hamsters [29,30,31]. In contrast to our findings that showed a combined IM-IN vaccination regimen failed to confer control of SARS-CoV-2 challenge and was poorly immunogenic, a chimp adenovirus delivered via the IN route showed superior immunogenicity and protection from SARS-CoV-2 challenge relative to IM vaccination in the hamster model [29]. Our results and other studies suggest that vaccine platform and route of delivery are important considerations for vaccine efficacy. Furthermore, our data show that despite significantly decreasing viral loads within the lungs of IM-only vaccinated hamsters, our vaccine failed to protect against lung pathology following SARS-CoV-2 challenge. Thus, in at least some vaccine contexts, decreased viral loads may not strictly correlate with improved lung pathology and in addition to effective control of viral replication, protection from severe COVID-19 may also require appropriate vaccine-induced immunity.

Our ELISA data showed that our IM prime-boost-boost regimen elicited significant anti-RBD antibodies. As the SARS-CoV-2 RBD is the target of neutralizing antibodies [32] we were surprised that this significant anti-RBD response measured by ELISA did not result in neutralizing activity against infectious SARS-CoV-2. This suggests our vaccine failed to elicit neutralizing antibody responses against SARS-CoV-2. These data further suggest that the decreased viral loads observed in the IM-only group occurred via mechanisms other than neutralizing antibody responses. Interestingly, multiple studies have reported conflicting claims, with some reporting that COVID-19 patients produce long-term neutralizing antibody responses while others suggest that these levels are actually low and wane quickly compared to non-neutralizing antibody titers [33]. While neutralizing antibodies may play a role in protection from disease, this does not exclude the importance of non-neutralizing antibodies which may inhibit virus by mediating antibody-dependent cellular cytotoxicity, fixing complement onto viral surface, or some yet to be uncovered mechanism [33]. Nonetheless, the non-neutralizing antibodies induced by our vaccine regimen did not appear to confer protection against SARS-CoV-2 induced pathology within the lungs. Although the IM-only group showed significant reduction in lung viral titers and weight loss, the similar lung pathology between IM-only and control vaccinated hamsters indicates that simply reducing viral replication may not be sufficient to prevent disease and that appropriate antibody and additional immune responses are required to prevent COVID-19. It is interesting to note that although we did not detect significant anti-RBD titers prior to challenge in the IM/IN combination group, we did observe a rapid and significant anamnestic response after challenge. Control vaccinated animals did not experience a similar increase in anti-RBD titers after challenge suggesting IM/IN vaccination did prime the host immune response to respond to SARS-CoV-2 challenge. An important limitation of our study is that we did not evaluate mucosal antibody titers, and systemic antibody titers in the serum may not fully represent the mucosal antibody response to the IN vaccination. Nevertheless, IM/IN vaccination did not protect against viral replication or virus induced pathology in the lungs suggesting this route was either poorly immunogenic or resulted in an inappropriate immune response.

Further, we cannot exclude a role for T-cells in protection and these responses could contribute to the decrease in viral loads seen in the IM-only group. Vaccine delivery vehicle and methods may be an important factor in inducing protective immune responses against SARS-CoV-2. In vivo electroporation vaccination strategies, such as CELLECTRA, have been reported to increase antigen delivery up to 1000-fold, in the best cases, compared to DNA delivery with no additional transfection methodology [34]. The CELLECTRA device, specifically, has been associated with increased memory T cell response not only in the COVID-19 vaccine study but also in a rhesus macaque simian immunodeficiency virus DNA vaccine study [35]. Thus, different modes of DNA vaccine delivery may induce varied immune responses and are important considerations in vaccine development to produce a protective immune response against SARS-CoV-2. Another DNA vaccine study, nCoV-S(JET), delivered 200 µg DNA by IM jet injection and was performed in Syrian hamsters with a prime-boost regimen of vaccinations 3 weeks apart. Similar to our study, this vaccination did not affect viral shedding in pharyngeal swabs but did reduce weight loss over the course of infection. In contrast to our study, the nCoV-S(JET) did induce neutralizing antibodies and decreased areas of consolidation and SARS-CoV-2 RNA labeling in areas of inflammation in the lung [36]. The distinct outcomes in our study with those of nCoV-S(JET) study suggest that neutralizing antibodies may play an important role in diminishing COVID-19 disease progression. The nCoV-S(JET) study further supports the likelihood that DNA delivery method is a significant factor in determining protective immune responses of vaccines.

Our study and others evaluating DNA-vaccines [36] supports the need for boosting to achieve significant antibody titer increase [27,36], an important public health consideration for deployment of vaccines against SARS-CoV-2 [27]. Alternative vaccine platforms may provide significant protection against COVID-19 after just a single vaccination. Hamsters and rhesus macaques vaccinated IM or IN with chimp adenoviruses expressing the SARS-CoV-2 spike protein, showed protection from SARS-CoV-2 after single immunizations [29,31,37]. Further, rhesus macaques vaccinated with a vesicular stomatitis virus encoding the spike protein were protected from SARS-CoV-2 challenge and development of lung pathology as soon as ten days after a single IM vaccination [28], indicating that some platforms may induce rapid protective immunity without the need for boosting. However, IN vaccination in the same study did not protect against virus challenge and resulted in increased lung pathology [28]. Interestingly, both IM and IN vaccination induced neutralizing antibody titers [28], suggesting that neutralizing antibodies are not always sufficient to protect against SARS-CoV-2 infection.

Cumulatively, the IM-only prime-boost-boost vaccine schedule significantly decreased SARS-CoV-2 lung viral loads, protected against weight loss, and elicited significant non-neutralizing antibody titers. In contrast, the same vaccine delivered via a combination IM/IN route failed to confer protection. Interestingly, despite reduced lung viral titers in some animals, lung pathology was not significantly impacted in any group, suggesting that lung viral loads may not strictly correlate with lung tissue damage and that proper vaccine-mediated immune responses are an important consideration for COVID-19 vaccines.

## Figures and Tables

**Table 1:** Histopathologic Lesions Summary. Numbers indicate the absolute number of animals from the respective vaccine group that fall into each category (lesion and severity) while the parentheses indicate overall percentage of animals from said vaccine group that fall in the category.

Vaccine Group.	Lesion	None	Minimal	Mild	Moderate	Severe
Control	Bronchiolitis	0	0	0	6 (100%)	0
	Interstitial Pneumonia	0	0	0	3 (50%)	3 (50%)
IM/IM/IM (IM-only)	Bronchiolitis	2 (17%)	0	3 (25%)	7 (58%)	0
	Interstitial Pneumonia	1 (8%)	0	2 (17%)	5 (42%)	4 (33%)
IM/IN/IN (IM/IN Combination)	Bronchiolitis	0	0	1 (8%)	11 (92%)	0
	Interstitial Pneumonia	0	0	0	5 (42%)	7 (58%)

**Figure A2:**
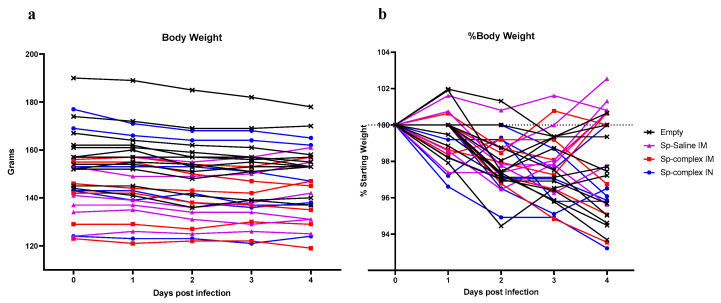
Prime-Boost Hamsters Detailed Body Weights. Prime-boost hamsters were divided into three spike-vaccinated groups receiving Sp_pVax1 IM vaccination of 100 µg DNA diluted in saline (Sp-Saline IM), IM vaccination of 50 µg Sp_pVax1 DNA complexed to in vivo delivery reagent (Sp-complex IM), and IN vaccination of 50 µg Sp_pVax1 DNA complexed to in vivo delivery reagent (Sp-complex IN). *n* = 12 for empty pVax1 group and *n* = 6 for Sp-vaccinated groups. Detailed body weight over course of infection for individual hamsters is shown as (**a**) exact body weight in grams or (**b**) percent body weight.

**Figure A3:**
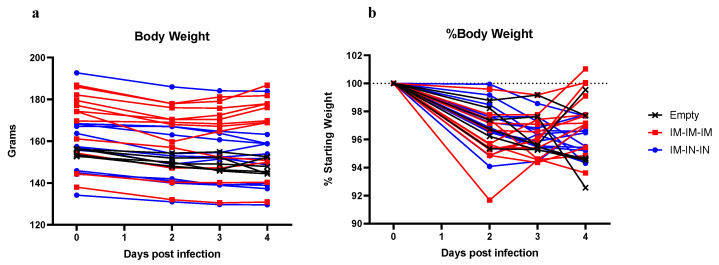
Prime Boost-Boost Hamsters Detailed Body Weights. Prime-Boost-Boost hamsters were divided into two spike-vaccinated groups receiving IM-IM-IM or IM-IN-IN vaccinations of 50 µg Sp_pVax1 DNA plasmid complexed to in vivo delivery reagent. *n* = 12 per group. Detailed body weight over course of infection for individual hamsters is shown as (**a**) exact body weight in grams or (**b**) percent body weight.

## Data Availability

All data reported in this study available upon reasonable request.
